# Malicious Vehicle Detection Using Layer-Based Paradigm and the Internet of Things

**DOI:** 10.3390/s23146554

**Published:** 2023-07-20

**Authors:** Abdul Razaque, Gulnara Bektemyssova, Joon Yoo, Aziz Alotaibi, Mohsin Ali, Fathi Amsaad, Saule Amanzholova, Majid Alshammari

**Affiliations:** 1 School of Computing, Gachon University, Seongnam-si 13120, Republic of Korea; arazaque@gachon.ac.kr; 2 Department of Computer Engineering, International Information Technology University, Almaty 050000, Kazakhstan; m.farhad@iitu.edu.kz; 3 Department of Computer Science, College of Computers and Information Technology, Taif University, P.O. Box 11099, Taif 21944, Saudi Arabia; 4 Computer Science Department, Wright State University, Fairborn, OH 45435, USA; fathi.amsaad@wright.edu; 5 Department of Cybersecurity, International Information Technology University, Almaty 050000, Kazakhstan; s.amanzholova@iitu.edu.kz; 6 Department of Information Technology, College of Computers and Information Technology, Taif University, P.O. Box 11099, Taif 21944, Saudi Arabia; m.alshammari@tu.edu.sa

**Keywords:** conventional neural networks, multi-label, IoT devices, deep learning, consortium blockchain technology, malicious vehicle detection

## Abstract

Deep learning algorithms have a wide range of applications, including cancer diagnosis, face and speech recognition, object recognition, etc. It is critical to protect these models since any changes to them can result in serious losses in a variety of ways. This article proposes the consortium blockchain-enabled conventional neural network (CBCNN), a four-layered paradigm for detecting malicious vehicles. Layer-1 is a convolutional neural network-enabled Internet-of-Things (IoT) model for the vehicle; Layer-2 is a spatial pyramid polling layer for the vehicle; Layer-3 is a fully connected layer for the vehicle; and Layer-4 is a consortium blockchain for the vehicle. The first three layers accurately identify the vehicles, while the final layer prevents any malicious attempts. The primary goal of the four-layered paradigm is to successfully identify malicious vehicles and mitigate the potential risks they pose using multi-label classification. Furthermore, the proposed CBCNN approach is employed to ensure tamper-proof protection against a parameter manipulation attack. The consortium blockchain employs a proof-of-luck mechanism, allowing vehicles to save energy while delivering accurate information about the vehicle’s nature to the “vehicle management system.” C++ coding is employed to implement the approach, and the ns-3.34 platform is used for simulation. The ns3-ai module is specifically utilized to detect anomalies in the Internet of Vehicles (IoVs). Finally, a comparative analysis is conducted between the proposed CBCNN approach and state-of-the-art methods. The results confirm that the proposed CBCNN approach outperforms competing methods in terms of malicious label detection, average accuracy, loss ratio, and cost reduction.

## 1. Introduction

The accurate detection of vehicles and the avoidance of malicious vehicles have become highly important. This task can be efficiently performed by utilizing a powerful and reliable object recognition model [1]. In recent times, researchers have focused on categorizing a single item into multiple categories during image recognition [2]. Therefore, the multiple labels provide excellent options for identifying the objects [3]. In multi-label classification, the presence of multiple labels signifies the fulfillment of a specific prediction [4]. The classification task in deep learning neural networks is considered significant because it enables an accurate prediction of class labels for hidden data [5]. Deep learning algorithms based on artificial neural networks (ANNs) have demonstrated exceptional prediction capabilities in solving complex tasks across various domains [6,7] including image classification [8], speech recognition [9,10], intrusion detection systems [11,12], and others. The convolutional neural network (CNN) can perform various operations on input images by repeatedly employing multiple layers to extract features efficiently and automatically [13,14,15]. In the case of multi-label classification, the classifier is trained separately, and the results are combined based on ranking [16,17]. However, predicting multiple conceptual labels remains a significant challenge. While CNNs are widely employed in modern applications as cutting-edge methods, their architectures lack a liability phase for each layer [18,19]. Unfortunately, artificial intelligence (AI) systems are susceptible to attacks at multiple levels, including input, architecture, and decision [20]. Consequently, the labels of objects in the image can be manipulated, leading to a decline in prediction accuracy [21].

Securing deep learning models is of utmost necessity [22]. To enhance output security and accuracy, it is essential to authenticate the layers of deep learning models [23]. However, privacy and security concerns exist since it is easy to steal deep learning models or change private training data [24,25]. Blockchain technology has gained widespread acceptance as a security solution as it ensures security, efficiency, and reduces fraud in CNNs [26,27,28]. Blockchain technology can prevent the manipulation of object labels by incorporating critical features such as security, authenticity, data privacy, and de-tracking [29]. To prevent manipulation within CNN architectures, the input parameters of each layer should be verified. Furthermore, the authenticity module ensures appropriate validation of outputs by comparing them across all layers. Data privacy and de-tracking features are employed to conceal sensitive information while also allowing the restoration of original data in the event of manipulation [30]. To address the challenge of assigning multiple labels to objects, a blockchain-enabled conventional neural network, named the consortium blockchain-enabled conventional neural network (CBCNN), can be developed to create a secure model and identify fraudulent labels, thereby combining the advantages of both approaches.

**Figure 1:**
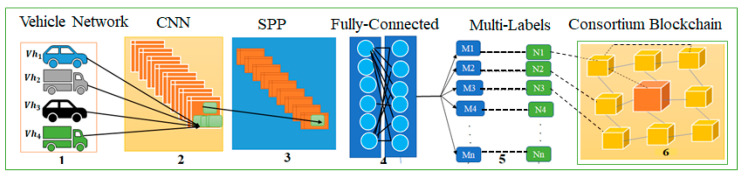
General architecture of CNN with blockchain technology using multi-labels.

The proposed CBCNN method is used for vehicle surveillance, specifically for detecting hostile vehicles. This approach is resistant to parameter manipulation attacks. Figure 1 illustrates the general architecture of CNN with blockchain technology using multi-labels.

### 1.1. Paper Contribution

The contributions of this article are summarized as follows:

▪ The layer-based paradigm is used to correctly detect and limit the malicious vehicle. This paradigm incorporates a CNN-enabled Internet of Things (IoT) model, geographic pyramid pooling, fixed-layer, and consortium blockchain technology. The primary objective is to achieve accurate application of the “malicious” label while enhancing accuracy and reducing the loss ratio.
▪ The CBCNN technique is employed to ensure tamper-proof protection against a parameter manipulation attack. Additionally, hostile vehicles are tracked and identified using multiple labels to prevent unauthorized entry.
▪ The consortium blockchain implements a proof-of-luck mechanism, enabling vehicles to conserve energy while providing correct information about the nature of the vehicle to the “vehicle management system”.
▪ The proposed CBCNN method is prototyped and experimentally assessed to illustrate its practicability and feasibility in real-time malicious vehicle detection scenarios.

### 1.2. Remaining Structure of Article

The remainder of the paper is organized as follows: Section 2 surveys related works as well as contemporary work on existing approaches. Section 3 demonstrates the system model of the proposed approach. Section 4 defines the problem formulation. Section 5 presents the proposed malicious vehicle detection process using a layer-based paradigm. Section 6 describes the experimental results and setup. Section 7 contains a discussion of the findings, including their benefits. Section 8 concludes with the findings of this article and future work.

## 2. Related Works

This section discusses the primary characteristics of existing methods. The secure convolutional neural network (SCNN) model is introduced as a solution to address potential attacks [31]. In this approach, each block contains a layer of a CNN model with their neighbors’ hash keys and public and private keys. The single point of failure issue is solved by introducing a multi-layer location sharing system based on blockchain [32], which can also be applied to dynamic situations. An accumulator is used to improve the efficiency of data refreshing and checking within the scheme. However, a continuous communication expense is associated with this scheme. The effectiveness of the proposed scheme is evaluated through a formal security study based on Real-Or-Random (ROR), and the experimental findings demonstrate its superiority over current techniques in terms of communication cost, computational cost, and efficiency. Ref. [33] addressed the security concerns arising from the transition to smart cities in an interconnected world. The blockchain model has been extensively analyzed as a potential solution to the security and privacy challenges of this new era. The interaction of blockchain with smart contracts is evaluated, including their implementation. The CNN is introduced to address the various types of attacks, such as model extraction and model inversion attacks, to preserve privacy; however, the proposed CNN method struggled to efficiently detect both types of attacks. Additionally, blockchain is employed in artificial intelligence to secure data sharing and automatically train models [34]. It is widely adopted to improve system security. Specifically, blockchain technology is used in conjunction with VGGFace deep neural networks to enhance the security and stability of face datasets, which are vulnerable to unauthorized access and tampering. To ensure data integrity, all pixel values are inserted into blocks before being sent to other nodes for access, significantly reducing data tampering.

Ref. [35] proposed a novel multi-model method combining deep neural networks and blockchain technology to characterize early-stage cancer from computed tomography (CT) scan images. The proposed model uses input data from each patient in the network to personify a specific health condition using low dose CT scan images. The deep neural network model personifies the report images, which contain information about the cancer nodules; this information is stored on the blockchain-distributed network for the patient. A privacy-preserving federated learning (FL) system, called BPFL, built on a secure blockchain, has been reported in the literature [36]. This system leverages blockchain as the fundamental distributed structure of FL. To accomplish ciphertext-level model aggregation and filtering, the Multi-Krum technology is enhanced and coupled with homomorphic encryption. The proposed approach can enable the verifiability of the local models while ensuring privacy protection. In this context, a privacy-preserving cross-domain authentication (PPCDA) system for vehicular ad hoc networks (VANETs) built on a secure blockchain is suggested [37]. The suggested approach combines group signing and blockchain technology to achieve its goals. The group signature technique is employed to provide limited privacy protection, whereas the suggested method relies on trustworthy information exchange to enable cross-domain verification among vehicles. Table 1 demonstrates the current work of existing approaches.

## 3. System Model

**Figure 2:**
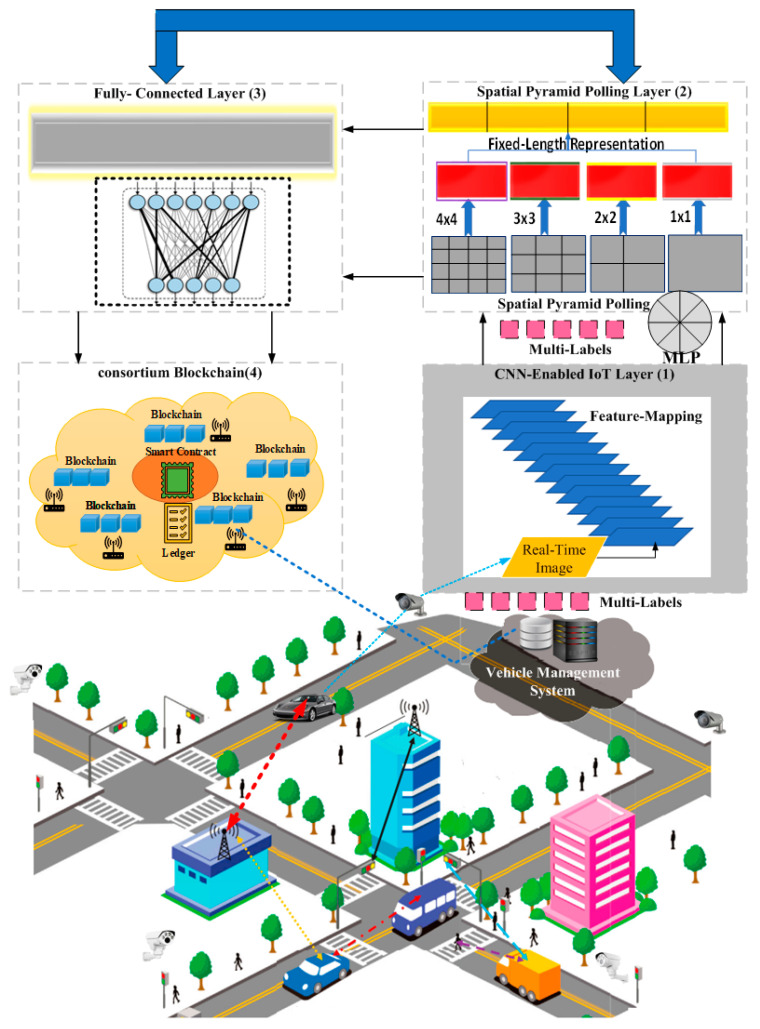
System Model for the proposed CBCNN.

Several measures are already in place to ensure vehicle security and safety, from design through manufacturing and operation. Our goal is to keep vehicles secure. The proposed system consists of four layers, each with a specific function in monitoring vehicles and storing information related to their malicious or non-malicious nature. The 4-layered paradigm is used in conjunction with IoT devices to detect malicious/non-malicious vehicles, which collectively execute efficient malicious/non-malicious activities ranging from monitoring to detecting the nature of the vehicle depicted in Figure 2. The system model supports the realistic scenario of traffic from Taepyeong-dong in Gyeonggi Province, South Korea, to Samseong-dong in Seoul, South Korea, which is a distance of 14.4 km. The realistic scenario is tested employing the layered-4 paradigm. Layer-1 is the CNN-enabled IoT layer in charge of monitoring vehicles and taking real-time pictures before applying the feature mapping technique to the obtained images; the feature mapping procedure represents specific qualities in the input image.

Layer 1 processes images from IoT devices (such as sensors, cameras, and network towers) positioned along the route and connected to the base station. When an image is detected, a sensor sends it to another sensor along with its ID and precise location. The sensor nodes that receive data create a list of adjacent nodes. From the collection of sensors, the sensor-enabled camera determines the ideal area for image capture. Because of this, IoT devices can connect via line-of-sight (LOS) despite a variety of physical impediments. As a result, the sensor node generates a list of its neighbors’ geolocations. The sensor node transmits the value of its cost to all its neighbors. Thus, images are successfully transmitted to CNN. Thus, the input image of the vehicle is convolved with one or more filters to yield numerous feature maps. Following this, the multilayer perceptron (MLP) model is applied to the output of layer 1 for the multi-label classification. Utilizing a multi-label approach for the vehicle provides more data to accurately identify its type. Therefore, labels are required as output for each input sample of the vehicle, and the outputs are required concurrently. The MLP transfers allocated multi-labels to Layer 2, known as spatial pyramid pooling (SPP), which overcomes the fixed-size limitation.

SPP collects local features from image layers of vehicles that have been classified based on their accuracy. SPP is the fundamental component used in effective classification and detection systems. The SPP and pooling approaches are coupled to allow neural networks to handle images of varying sizes as input. Pooling allows for the collection of data from neighboring space bins. The size of the space bins remains constant regardless of the size of the vehicles’ input images, while the size of the images is positively connected with it. Furthermore, the SPP layer aggregates the features and generates fixed-length outputs that are fed into the fully connected layers. This information aggregation occurs at a deeper level in the network structure between the convolutional layers and fully connected layers, thereby eliminating the need for cropping or warping the image of the vehicle.

Layer 3, the fully connected layer, compares the similarity of the input and categorizes the input label. The completely connected layer demonstrates a high capability for feature fusion. A fully connected layer mixes the input with a weight matrix and adds a bias vector. A fully linked layer’s neurons communicate with all neurons in the preceding layer. The neuron in fully connected layers linearly changes the input vector using the weight matrix. After that, a non-linear activation function is used to apply a non-linear change to the result in order to detect the exact vehicle.

The fully connected layer translates the vehicles’ generalized graphical representation onto the given sample space. Layer 3 oversees sending the label information from the target loss function to the preceding layer.

Layer 4 utilizes a consortium blockchain to identify malicious vehicles and prevent potential attacks. The consortium blockchain also safeguards data privacy. Layer 4 ensures that the distributed shared databases remain up-to-date. The consortium blockchain achieves two objectives: (i) reducing latency while maintaining data privacy, and (ii) enabling quick data transmission between vehicles. During communication establishment, vehicles undergo verification to prevent attackers from maliciously utilizing resources to disrupt the lawful message process and thereby compromise vehicle identification. The consortium blockchain employs a proof-of-luck method that enables vehicles to conserve energy. Vehicles participating in communication and receiving the most votes can seize control of the entire vehicular network. Consequently, the selected vehicles are rewarded for their participation in the validation process and sequential block construction. If a vehicle fails to create its assigned block, the task is passed on to the next vehicle, and the other vehicles decide to replace it with a new vehicle (witness). This solution also effectively addresses latency issues and provides robust protection against duplicate pending attacks through the immunity of the proof-of-luck mechanism. Finally, the state of the vehicle is presented on the vehicle management system based on the assessment conducted using consortium blockchain technology. Figure 1 depicts the system model of the proposed CBCNN.

## 4. Problem Formulation for Cost Reduction Using Multi-Labels

The problem can be formulated by generating multi-labels (ml) to be delivered to vehicles v within a certain range. The vehicles must be monitored in all directions to ensure that each vehicle receives the appropriate label. Therefore, the multi-label assignment process can be determined as:

(1) $$V_{i}\left(v,O\right)=\{\begin{array}{c} 1\;\;\;\;\;\;\;\;\;\;\;\;\;\;\;\;\;if\;V_{i}\leq V_{p}\\ 0\;\;\;\;\;\;\;\;\;\;\;\;\;\;\;\;Otherwise \end{array},$$

where v denotes the vehicle, $V_{i}$ denotes the number of vehicles in the network, and $V_{p}$ denotes the position of the vehicle.

The multi-label delivering intensity, $D_{I\left(ml\right)}$ is an important variable that helps to reach all of the vehicles within time, which can be determined as:

(2) $$D_{I\left(ml\right)}=max\sum_{i=1}^{V_{t}}V_{i}\left(v,O\right)$$

Equation (2) indicates that the intensity and the maximum number of multi-labels given to vehicles should be significantly higher. This is particularly important for multi-labels because accurate identification requires significantly closer spacing. As the distance increases, the monitored measurements become attenuated and deviate from the initial values. To prevent malicious vehicles, a high-detection-density multi-label detection system is proposed.

(3) $$V_{i}\left(V,O\right)=\frac{{\left(cos\theta \left(T_{1},T_{2}\right)\right)}^{\partial }}{r},\;\;\;$$

where $cos\theta \left(T_{1},T_{2}\right)$ represents the values for two tuples used to locate the vehicles, r represents the distance between the sending and receiving vehicles and ∂ indicates the associated reduction.

The proposed approach cannot simultaneously encompass the entire area where all vehicles are moving to distribute the multi-labels. Therefore, it is critical to divide all potential exposure routes by setting coordinates at the starting point, which is regarded as one end and symbolizes where a specific vehicle has begun working with multi-labels. The opposing extremity of the exposure route is then placed on the precise opposite side. As a result, a high-dimensional probability $H_{pr}$ may be used to define the route of the vehicle on the pathway given by:

(4) $$H_{pr}\left(I_{p},\Gamma \right)=\int_{i=0}^{ml_{t}}I_{p}\left(R_{d}\right)dl,\;\;\;$$

where $I_{p}$ represents the initial position of the vehicle relative to the robot, Γ determines all corresponding pathways, $ml_{t}$ denotes the total number of multi-labels to be delivered to vehicles, and $R_{d}$ indicates a random distance between the vehicle and the multi-labels.

High-dimensional probability represents a non-linear process that can only be resolved by splitting all routes under consideration into several sub-interval points between vehicles and labels.

(5) $$H_{pr}\left(I_{p},\Gamma \right)=min\sum_{i=0}^{ml_{t}}I_{p}\left(R_{d}\right)\Psi ,\;$$

where Ψ is the sub-interval point in the pathways.

The sub-interval points should be minimized to achieve the best data aggregation method. This technique is necessary for the multi-labeling of vehicles. When two paths, such as distribution and destination, are correctly defined, this method works flawlessly. However, if a specific vehicle action is moved to the opposing side, a minimal degree of familiarity is achieved. At these two node locations, where vehicles are connected to monitor the vehicle automation process, it is critical to consider the weight of the multi-label. The weight of a multi-label $ml_{w}$ can be determined as:

(6) $$ml_{w}=\overset{ml_{t}}{\underset{i=0}{\sum }}ml_{w1}+ml_{w2},\;.\;.\;.\;,+ml_{wi}$$

Therefore, $ml_{w}=1$.

While each vehicle travels, different weights of the multi-labels are continuously generated. Consequently, multiple label weights are added. The transmission of data to the correct vehicles should also be used to establish distinct vehicle monitoring periods for the data collection process. This variable time for moving the vehicle $t_{v}$ can be determined as follows:

(7) $$t_{v}=\left(V_{t}+Td_{r}+t_{b}\right),\;\;\;$$

where $V_{t}$ indicates the total number of vehicles, $Td_{r}$ represents the data transmission rate, and $t_{b}$ denotes the buffering time of loading data.

Additionally, the cost of each multi-label should be established to evaluate the operation of vehicles. This is critical because each multi-label has a distinct weight and requires various motions to efficiently move and transmit a large quantity of data. Therefore, the cost of producing multi-label $ml_{g}$ to transport the vehicles can be calculated as follows:

(8) $$ml_{g}\left(min\right)=\overset{ml_{t}}{\underset{i=0}{\sum }}\left(ml_{wi}\times ϣ\right)$$

where ϣ represents the decision-making process and $ml_{wi}$ represents the weight of the multi-label.

The desired outcome of cost reduction $C_{R}$ of the proposed CBCNN approach can be mathematically expressed by integrating Equation (1) through Equation (8):

(9) $$C_{R}=min\overset{ml_{t}}{\underset{i=0}{\sum }}V_{i},H_{pr}\left(I_{p},\Gamma \right),max\overset{ml_{t}}{\underset{i=1}{\sum }}V_{i}\left(v,O\right)\;$$

According to the anticipated results of the proposed CBCNN, the cost is reduced, whereas the vehicle detection intensity is increased. This indicates that the use of multi-label should maximize detection strength while minimizing cost and sub-interval location.

## 5. Proposed Malicious Vehicle Detection Process Using Layer-Based Paradigm

The CBCNN is used to identify and restrict malicious vehicles. The proposed model consists of four layers that work together to aid in vehicle identification and the prevention of hostile vehicle activities. One of the key strengths of this paradigm is its use of multiple labels to achieve accurate detection. The proposed CBCNN makes use of the following traits:

▪ Multi-label classification for vehicles.
▪ CNN-enabled IoT layer modeling for vehicles
▪ Spatial pyramid polling layer modeling for vehicles
▪ Fully connected layer modeling
▪ Consortium blockchain technology modeling for vehicles
▪ Multi-label modeling for vehicles

### 5.1. CNN-Enabled IoT Layer Modeling for Vehicle

CNNs achieve cutting-edge performance by effectively dealing with multiple labels. This success can be attributed to its ability to learn feature characteristics, unlike traditional methods that rely on hand-engineered features. The CNN is particularly useful for handling vehicles using multiple labels in sequential data. It captures sequence dynamics through network cycles and stores positional information that represents data from a randomly extended context window. The authentication framework was created to support multi-label classification for vehicles. Given that it is possible that most inputs have been corrupted, the authentication framework consists of a short preamble of multiple symbols preceding the multi-label authentication process. These symbols have fixed periods and frequencies; the proposed framework can detect these brief preambles and pass them on to the classifier. Two or more labels are generated as input to the classifier. If the multi-labels are sent as the number of samples $S_{n}$ as input to the classifier, the length of each input sample becomes $2S_{n}$. Thus, the number of the classifier units $C_{n}$ can be determined in each layer β as:

(10) $$C_{n}=\lceil \frac{\beta }{S_{n}}\rceil \;\;\;$$

The CNN begins processing the images received from IoT devices (such as sensors, cameras, and network towers) installed around the road, that are connected to the base station. A sensor detects the image and communicates it with another sensor along with its ID and exact location. The receiving sensor nodes compile a list of their neighbor nodes $S_{ne}$. The sensors are grouped together, and the sensor-enabled camera fine-tunes the location for image capture. This capability allows IoT devices to establish a line-of-sight (LOS) connection despite various natural limitations. Consequently, the sensor node generates a list of its neighbor nodes $S_{ne}^{L}$, along with their geo-locations. The cost value of the sensor node is broadcast to all neighbors. The sensor nodes are organized into a polygon structure, and the area of the polygon $A_{P}$ is determined as follows:

(11) $$A_{P}=\frac{1}{2}\overset{K_{t}}{\underset{i=0}{\sum }}\left(GL_{i}^{P}*GL_{i+1}^{Q}\right)-\left(GL_{i+1}^{P}*GL_{i}^{Q}\right)\;\;\;\;$$

where $GL_{i}^{P}$ denotes the lists of geo-locations of sensor nodes positioned at the polygon edge obtained by the *x*-axis; $GL_{i+1}^{Q}$ denotes the lists of geo-locations of sensor nodes positioned at the polygon edge obtained by the *y*-axis; and $K_{t}$ denotes the total number of sensor nodes. Therefore, the centroid $C_{P,Q}$ can be determined as follows:

(12) $$C_{P,Q}=\frac{1}{6A_{p}}\overset{K_{t}}{\underset{i=0}{\sum }}\left(GL_{i}^{P}+GL_{i+1}^{P}\right)\left(GL_{i}^{P}*GL_{i+1}^{Q}\right)-\left(GL_{i+1}^{P}*GL_{i}^{Q}\right)\;$$

And

(13) $$C_{Q}=\frac{1}{6A_{p}}\overset{K_{t}}{\underset{i=0}{\sum }}\left(GL_{i}^{Q}+GL_{i+1}^{Q}\right)\left(GL_{i}^{P}*GL_{i+1}^{Q}\right)-\left(GL_{i+1}^{P}*GL_{i}^{Q}\right)\;$$

Merging the *x*- and *y*-axes together yields the following equation:

(14) $$C_{P,Q=}\left(C_{P},C_{Q}\right)\;\;\;$$

The probability for each node to become the cluster head $Pr_{ch}$ is determined using the following calculation:

(15) $$Pr_{ch}=Max\left\{\left(1-\frac{\epsilon_{r}}{S_{r}}\times n_{f}\right),K_{min}\right\}\;\;$$

where $\epsilon_{r}$ is the distance between two neighboring nodes, $S_{r}$ is the radius of the sensor, $n_{f}$ is the normalization factor, and $K_{min}$ is the minimum threshold of the sensor node.

The distance between the central node $\left(K_{c}\right)$ and the current node $\left(K_{cu}\right)$ is determined using the concept of Euclidean distance $P_{\left(K_{c}\right),\left(K_{cu}\right)}$, which is calculated as follows:

(16) $$P_{\left(K_{c}\right),\left(K_{cu}\right)}=\sqrt{{\left(\left(K_{c}\right)P-\left(K_{cu}\right)P\right)}^{2}+{\left(\left(K_{c}\right)Q-\left(K_{cu}\right)Q\right)}^{2}}$$

Thus, the image collection process $I_{C}$ can be calculated prior to transmission to the CNN-enabled IoT layer.

(17) $$I_{C}=\left(\partial_{i}\left(I\right),\left(CH\right){ϣ}_{i}\left(I\right)\right)+\left(\left(CH\right){ϣ}_{f}\left(I\right),\partial_{f}\left(I\right)\right)+\overset{I_{t}}{\underset{i=0}{\sum }}\left\{\left(CH\right){ϣ}_{i}\left(I\right)\right\}i,\left\{\left(CH\right){ϣ}_{i}\left(I\right)\right\}i+1\;$$

where, $\partial_{i}\left(I\right)$ represents the starting-image collection location to the first location of the cluster head $\left(CH\right){ϣ}_{i}\left(I\right)$, ${ϣ}_{f}\left(I\right))$ is the final location of the cluster head to the ending image collection location $\partial_{f}\left(I\right)$, and $I_{t}$ is the total number of images.

### 5.2. Spatial Pyramid Polling Layer Modeling for Vehicle

An SPP network processes an input feature map using different filtering or pooling rates, potentially creating a range of practical fields of vision for the vehicles. Before combining feature maps, a normalization procedure is necessary. Before concatenation, batch normalization is employed at each filter to account for the relative differences in feature values across layers. SPP extracts local characteristics from image layers of vehicles grouped based on the degree of accuracy. SPP is consistently the primary element employed in effective classification and detection systems. The SPP and pooling techniques are combined to enable neural networks to handle input images of various sizes. Pooling enables the acquisition of information from nearby space bins. The size of the space bins remains constant regardless of the size of the input images of the vehicles, while the size of the images is positively correlated with it; this differs from standard deep networks in which the number of sliding windows is fixed regardless of the size of the input images. Multi-scale SPP layers are employed in place of max-pooling layers to enable deep networks to adapt to input images of vehicles of any size. The response of the filters is randomly gathered in each space bin. The outputs of SPP are $N_{f}$-dimensional vectors with a fixed length for N bins, where f is the number of filters used for the vehicles. Algorithm 1 illustrates the SPP process for vehicles.
**Algorithm 1:** Spatial pyramid polling modeling for vehicle**Initialization:** {$V_{im}$: Vehicle image; $C_{f}$: Fixed size input constraints; $O_{f}$: Fixed size output; Γ: Features; Ŕ: Removal of fixed size input constrains; τ: aggregation; $C_{a}$: Adjustable-sized constraints}**Input:** {$C_{f}$}**Output:** {$O_{f}$}**Set**$V_{im}$**If** Γ$≅C_{f}$ then**Process** $Ŕ\to C_{f}=C_{v}$**Assign** $C_{a}\;\to C_{NN}$**end-if****Perform** $\tau ↔V_{im}=O_{f}$ **Forward** $O_{f}\to F_{l}$**Return step-4**

Step 1 initializes the variables that will be utilized. Steps 2 and 3 supply the input and output, respectively. Step 4 shows the assignment of the vehicle image to the CNN-enabled IoT layer. Step 5 determines if the extracted image features have fixed-sized input, as the CNN-enabled IoT layer does not require fixed-sized input. Steps 6–8 describe the method of removing fixed-sized restrictions to create adjustable-sized input constraints that are compatible with CNN. Step 9 performs the aggregation procedure on the vehicle image obtained from CNN, converting it into a fixed-sized output suitable for the fixed-sized layer. Step 10 demonstrates the method for passing fixed-sized constraints to a fixed-sized layer. Finally, Step 11 initiates the image vehicle procedure described in Step 4.

SPP receives an input Ī from the previous layer for feature mapping, which is determined as

(18) $$Ī=Cf_{o}\left({Ī}_{1},{Ī}_{2},\;.\;.\;.\;,Ī-1\right),\;$$

where, $Cf_{o}$ is the composite function operation in the SPP layer, and concatenation occurs within every feature layer.

The time and space complexities of the spatial pyramid polling modeling algorithm for vehicles are O (log n).

Each SSP process produces a fixed-length feature image of the vehicle. V represents the vector level of SPP, which has a single feature map of any length. Therefore, the vector-level SPP process for the vehicle $V_{SPP}$ can be determined as follows:

(19) $$V_{SPP}=\overset{V}{\underset{i=1}{\sum }}G_{i}^{2}\;\;,\;$$

The total feature length produced by SPP when applied to the standard block $C_{b}$ can be determined as:

(20) $$SPP_{\left(C_{b}\right)}=V_{SPP}\times \nabla T_{fm},\;$$

where $\nabla T_{fm}$ is the total feature mapping process and i is the index of the SPP grid $G_{i}$.

The purpose of SPP is to reduce the spatial dimension of feature maps, thereby reducing the number of training parameters and computing complexity.

### 5.3. Fully Connected Layer Modeling for Vehicle

A fully connected layer for the vehicle combines the input using a weight matrix and adds a bias vector. All neurons in a fully connected layer communicate with all neurons in the preceding layer. Through the weight matrix, the neuron in fully connected layers linearly transforms the input vector. A non-linear activation function $n_{f}$ is then employed to apply a non-linear transformation to the result for detecting the exact vehicle Ύ, which is given as:

(21) $$Ύ=n_{f}\left(\overset{h_{t}}{\underset{i=1}{\sum }}M_{w}\times V_{in}\right)+B_{t}\;\;\;$$

where $h_{t}$ is the total number of hidden layers, $M_{w}$ is the weight of the matrix, $V_{in}$ is the input vector, and $B_{t}$ is the bias term.

The fully connected layer maps the generalized graphical representation of the vehicles onto the specified sample space. The fully connected layer is responsible for transmitting the label information for the categories from the target loss function to the preceding layer. A fully connected layer is commonly used to establish a successful mapping between multi-label information and the feature space of vehicle images, which can be determined as follows:

Let $V_{im}$ be the image of the vehicle, and ϐ is the kernel at the fully connected layer. The output image $O_{im}$ can thereby be obtained after the operational process.

(22) $$O_{im}\left[c_{in\left(x\right)},c_{in\left(y\right)}\right]=\overset{w}{\underset{p=1}{\sum }}\overset{h}{\underset{q=0}{\sum }}V_{im}\left[p,q\right]ϐ\;\left[\left(c_{in\left(x\right)}-p\right),\left(c_{in\left(y\right)}-q\right)\right]\;$$

where, $c_{in\left(x\right)}$ and $c_{in\left(y\right)}$ are the coordinates of the input image, p and q are the coordinates of the layer, w is the weight of the vehicle image, h is the number of hidden layers required for processing the image at the fully connected layer, $A_{Ne}$ is the activity of the neuron, $K_{t}$ is the total number of kernels in the fully connected layer; e,  h,  Б, and *Ϋ* are the values of the hyperparameters established using the validation set.

It is crucial for the mapping procedure to determine the normalization outcome for the executed action. Thus, the normalization outcome $N_{o}$ is as follows:

(23) $$N_{o}=A_{Ne}\times {\left\{Б+h\overset{min\left(K_{t}-1,\;i=\frac{e}{2}\right)}{\underset{j=max\left(0,\;i-\frac{e}{2}\right)}{\sum }}{\left(A_{Ne}{}_{\left(j\right)}\right)}^{2}\right\}}^{Ϋ}\;$$

A fully connected layer is commonly used to establish a successful mapping between multi-label information and the feature space of vehicle images. Therefore, the probability of the sampling $Pr\left(S_{in}\right)$ can be determined as:

(24) $$Pr\left(S_{in}\right)=e^{\theta .S_{in}}/\overset{V_{tim}}{\underset{V_{im}=1}{\sum }}e^{\theta .S_{in}}$$

where, $V_{tim}$ is the total number of images for the vehicles; $S_{in}$ is the input sample.

### 5.4. Consortium Blockchain Technology Modeling for Vehicle

**Figure 3:**
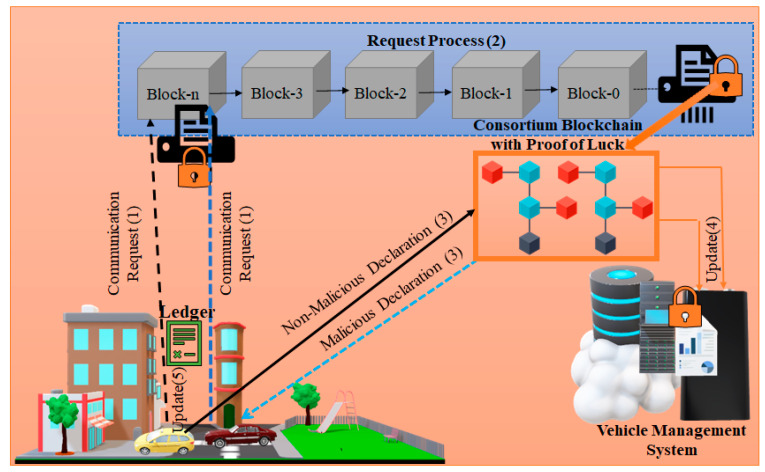
Malicious/Non-malicious vehicle detection process using consortium blockchain technology with a proof-of-luck mechanism.

The consortium blockchain is used to detect malicious and non-malicious vehicles depicted in Figure 3. It serves the purpose of facilitating the exchange of multiple labels and modifying information to ensure process, scalability, and accountability. Combining the advantages of private and public blockchains with those of CNN, the consortium blockchain differentiates itself by alleviating network burdens in scenarios involving a small number of vehicles. It is important to consider the number of vehicles connected to the consortium blockchain technology; however, there is the risk of capturing or modifying the multi-labels, which can lead to the detection of incorrect vehicles instead of the suspected ones. Therefore, blockchain technology makes it easier to restrict label alterations.

Algorithm 2 describes the process of detecting malicious vehicles.
**Algorithm 2:** Malicious vehicle detection process using multi-labels based on consortium blockchain**Initialization:** {V:Vehicle; Vm; Malicious vehicles; Lm: Multi-Label; E: Encoder; C: Classifier; Lmul: Multi-layers; $Lm^{-}$: Encoded Multi-Label; $B_{ts}$: Blockchain technology server; D: Decoder; $Lm_{B}$: Multi-label block; $Lm_{t}$: Total multi-labels; Fm: Feature matcher}**Input:** {V, Lm, $m_{B}$}**Output:** { Vhm}**Set** $Lm=E≅Lm^{-}$**Process** $E\left(Lm^{-}\right)\to C$**Forward** $C\left(Lm^{-}\right)\to Lmul$**Calculate** $Lmul\left(Lm^{-}\right)\to D⊕\left(Lm^{-}\right)=Lm\in C$**Forward** $Lm\in C\;\to B_{ts}⌅\;Lm_{B}$**Set** $Lm_{B}=B_{ts}$**If** $Lm_{B}=M_{B}$ then**Block** $Lm_{B}$**else** $Lm_{B}\;\neq M_{B}$ then**Allow** $Lm_{B}$**End-else****End-if** **While** $\left(Lm_{B}\;\leq Lm_{t}\right)$Store $Fm=m_{B}++$**If** Fm=∃ V then//* malicious features of the vehicle are matched**Set** V=Vm**else** Fm≠∃ V then//* malicious features of the vehicle are not matched.**Set** V≠Vm**End-else****End-if****End-while**

The variables are initialized in step 1. Steps 2 and 3 represent the input and output, respectively. Steps 4 and 5 encode the multi-labels and send them to the CNN classifier for action. Steps 6–9 involve the classifier sending the multi-labels to the hidden layers. Once the packets reach the decoder, the decoder converts the encoded multi-labels to the original multi-labels and sends the multi-labels (multi-label blocks) to the blockchain technology. Steps 10 and 11 start the process of detecting malicious multi-label blocks; if any malicious blocks are identified, they are restricted from further propagation. Steps 12 and 13 illustrate that if the block is not identified as a malicious block, it is permitted to proceed. The suspected vehicles are identified in steps 16–21. The features of the malicious blocks are stored in the feature detection component, and these features are compared with those of the vehicle. If the features match, the vehicle is classified as a suspect vehicle; otherwise, it is classified as a legitimate vehicle.

The blockchain server for further processing, as specified in the algorithm:

(25) $$Lm\in C\;\to B_{ts}⌅\;Lm_{B}\;$$

If any vehicle is suspected, its multi-labels Lm are added to the multi-label block $Lm_{B}$. After checking all the multi-labels, these $Lm_{B}\in M_{B}$ are blocked or restricted from further transmission, and v is assigned as Vs. This process blocks each suspected vehicle individually from transmitting any message, allowing the identification and storage of attacker patterns through suspicious blocks in $B_{ts}$ for future reference. Consequently, consortium blockchains are utilized to secure the CNN model.

**Corollary** **1.** *Blockchain enhances the capability of neural networks to detect malicious labels more efficiently*.

**Corollary** **2.** *Blockchain ensures the secure transmission of data between CNN layers.*

**Hypothesis** **1.** *If the feature detection component is kept alongside multi-labels, the likelihood of detecting incorrect vehicles is reduced*.

**Proof.**  The feature detection component successfully detects the suspected vehicle by examining the features of all blocks. Equation (26) represents three types of vehicles based on labels: neutral, suspected, and legitimate.

(26) $$V_{t}=\left\{V_{s},\;V_{l},\;V_{ne}\right\}\;$$

Here $V_{t}$ represents vehicle types, where $V_{ne}$ is a neutral vehicle not yet detected or classified, $V_{s}$ is a suspected harmful vehicle, and $V_{l}$ is a legitimate vehicle. The vehicle dataset $V_{D}$ can be defined as:

(27) $$V_{D}=\left\{V_{1},\;V_{2},\;\ldots ,V_{n}\right\},$$

where $V_{D}$ represents the vehicle dataset and $V_{1},\;V_{2},\;\ldots ,V_{n}\;$ denote the feature vectors in the dataset.

The behavior of the data is denoted as $B_{V}$; this behavior illustrates the data relationships and dependencies based on weights. The behavior of a sample $B_{SD}$ belonging to the suspected class $C_{s}$ is defined as follows:

(28) $$B_{SD}=B_{V}\;\times \;C_{s}$$

The behavior of the data $B_{V}$ can assume values of 1 or 0, representing suspected or legitimate behavior, respectively. If a sample contains 1, the label is suspected; if it contains 0, the label is legitimate. After the encoding and decoding processes, the multi-labels are transferred and stored in blocks $Lm_{B}$, and the features of these blocks are stored in the feature matcher Fm. The feature matcher compares the features of each block based on the sample behavior $B_{SD},\;\mathrm{as}\;\mathrm{shown}\;\mathrm{in}\;\mathrm{Equation}\;\left(28\right)$. If a suspected vehicle is present, the features of malicious blocks are stored in the feature matcher Fm, and the vehicle is classified as suspected. Storing only multi-labels (containing symmetric, asymmetric, and hash keys of neighbor nodes) has been shown to potentially cause alteration; thus, incorrect vehicles can be detected as suspect. Due to the higher accuracy of CBCNN, the feature matcher component performs an additional check to prevent incorrect vehicle detection.

There are four circumstances for Algorithm 2 that must be avoided since they may result in the hypothesis being rejected. The following are the situations under which a hypothesis is rejected.

▪ If the feature detection component is not kept alongside multi-labels, the likelihood of detecting incorrect vehicles is increased, and the hypothesis is rejected.
▪ If the threshold value set for the behavior of the data $B_{V}$ is negative or higher than one, then the hypothesis can be rejected.
▪ The features of the malicious blocks are set as null values in the feature detection component, and the hypothesis is rejected.
▪ The likelihood that the procedure of assigning the multi-labels to a vehicle would result in a type I error (rejecting the null hypothesis when it is actually true).

Moreover, our main objective is to accept the hypothesis in order to correctly detect the malicious/suspected vehicles. □

**Definition** **1.** *The feature matcher mechanism reduces the true negative rate (true label adjudged as false label) of the CBCNN classification algorithm*.

**Corollary** **3.** *Using CBCNN, the feature matcher reduces the likelihood of incorrect malicious labels.*

**Theorem** **1.** *The vehicle* v *provides labels* $\left\{l_{1},l_{2},\;.\;.\;.\;,\;l_{v}\right\}$*, the classifier can retrieve and protect detected multi-labels.*

**Proof.**  Assuming that a classifier receives $\left\{l_{1},l_{2},\;.\;.\;.\;,\;l_{v}\right\}$, where we assume that the identity of the label is the $l_{id\left(j\right)}=j$ and we mark $l_{1}=l$. According to the Lagrange interpolation theorem L(x), there exists a unique polynomial l of degree at most v−1 that passes through vehicles $\left\{\left(1,l_{1}\right),\left(2,l_{1}\right),\ldots ,\left(v,l_{1}\right)\right\}$, which can be expressed as:

(29) $$L\left(x\right)=\overset{v_{t}}{\underset{i=1}{\sum }}l.j\left(v_{i}\right)\;\;\;\;$$

Hence, $v_{j}=\prod 1\leq t\leq v;\;t\neq j\;\left(\frac{l-t}{j-t}\right)$, where, t is the time to deliver the label to the vehicle.

Hence, l can be derived from the given proposition to process the multi-labels $m_{l}$ with probability $p_{i}$, which may be expressed as follows:

(30) $$m_{l}=\overset{l_{t}}{\underset{i=1}{\sum }}p_{i}\;\;\;$$

The steady coefficient of l is the sum of the steady coefficients of every $P_{i}$, which is developed through the polynomials ${\left\{P_{i}\right\}}_{i=\left(1,m\right)}$, encompassing the ciphertexts.

The sum of the steady coefficients for each $P_{i}$ created by the polynomials is known as the steady coefficient of multi-label $m_{l}$. This applies to the entire set of ciphertexts.

(31) $$m_{l}=\overset{l_{t}}{\underset{i=1}{\sum }}C_{i}\;$$

where $C_{i}$ represents the contents of the label l and $l_{t}$ denotes the total number of labels.

The contents of each multi-label can be determined as follows:

(32) $$C_{i}=\left(r_{i}G,M_{i}+r_{i}\left(\;lG\right)\right)\;\;$$

Herein, $M_{i}$ is the message, $r_{i}$ is a random number, and G is the random number generator.

Consequently, the multi-label consists of contents, a random number, and a random generator to safeguard the multi-label delivered to the vehicle, which can be computed as follows:

(33) $$m_{l}=\overset{l_{t}}{\underset{i=0}{\sum }}C_{i}=\left\{\left(\overset{l_{t}}{\underset{i=1}{\sum }}r_{i}\right)G+\overset{l_{t}}{\underset{i=1}{\sum }}M_{i}+\left(\overset{l_{t}}{\underset{i=1}{\sum }}r_{i}\right)lG\right\}\;$$

Thus, the security of multi-label is computed using the random number generator and label generator.

(34) $$m_{l}=\left(\overset{l_{t}}{\underset{i=1}{\sum }}r_{i}\right)lG\;\;$$

To protect the multi-label, the reverse mapping function $M_{f}$ and the homomorphic characteristic Γ are utilized.

(35) $$m_{l}=\overset{l_{t}}{\underset{i=1}{\sum }}{\left(M_{f}\right)}_{i}*\Gamma \;\;\;$$

Therefore, an elliptic curve ∀ε is employed to protect the multi-label. The group of elliptic curve elements $\forall \varepsilon =\left(\varepsilon_{1},\;\varepsilon_{2}\;,\;.\;.\;.\;,\;\varepsilon_{N}\right)$, which form an Abelian cluster, is mapped into the entire plaintext, which can be determined as:

(36) $$m_{l}=\overset{l_{t}}{\underset{i=1}{\sum }}{\left(T_{p}\right)}_{i}+\forall \varepsilon \overset{l_{t}}{\underset{j=1}{\sum }}{\left(\varepsilon_{1},\;\varepsilon_{2}\;,\;.\;.\;.\;,\;\varepsilon_{N}\right)}_{j}\;\;$$

Furthermore, this method provides proof for protecting the multi-label.

The plaintext $T_{p},$ representing the multi-label given to a vehicle, has been observed to be secured. It should also be assumed that the multi-label $m_{l}$ is protected against a brute force approach by assigning the originator point to itself, unless a number of summations s that creates $m_{l\;}$ are sought. This technique is suitable for multi-label protection. □

### 5.5. Multi-Labels Modeling for Vehicles

To determine the potential associations between objects of different classes, we calculate the classification accuracy of multi-labels. Errors in classification can lead to inaccurate driving actions, as the driver’s behavior towards pedestrians should differ significantly from that towards vehicles. The accurate classification denoted as $A_{c}$ can be defined as:

(37) $$A_{c}=\left\{\left(P_{r},\varphi \right)\epsilon T_{p\left(gc\right)}⋀\;Class\left(P_{r}\right)=Class\left(\varphi \right)\right\},\;$$

where gc represents the generic class.

The accurate classification $A_{c}$ for a specific class $C_{p}$ can be determined as follows:

(38) $$A_{c}\left(C_{p}\right)=\left\{\left(\left(P_{r},\varphi \right)\epsilon T_{p\left(C_{p}\right)}\right)⋀\;Class\left(P_{r}\right)=C_{p}\right\}\;$$

Therefore, the overall classification accuracy for the multi-label is denoted as $A_{o\left(ml\right)}$ can be computed as follows:

(39) $$A_{o\left(ml\right)}=\frac{\left|A_{c}\right|}{\left|T_{p}\right|}\;\;$$

Furthermore, the classification accuracy for a specific class within the multi-label $A_{o\left(C_{p}\right).\left(ml\right)}$ can be determined as follows:

(40) $$A_{o\left(C_{p}\right).\left(ml\right)}=\frac{\left|A_{c}\left(C_{p}\right)\right|}{\left|T_{p\left(C_{p}\right)}\right|}$$

The final classifier produces an output that contains all the label information, which is then transmitted to the final layer connected to blockchain technology. In the proposed approach, we utilize multiple labels to identify vehicles. To successfully deliver the multi-labels, the vehicles must provide accurate location information. Thus, the precise location of the vehicle, $V_{pl}$, can be calculated as follows:

(41) $$V_{pl}=\left\{V_{\left(ina\right)}\;,V_{av},V_{s},\;V_{l},\;V_{ne}\right\}$$

Herein,$\;V_{s}$ represents the suspected vehicle, $V_{ne}$ represents the neutral vehicle, $V_{l}$ represents the legitimate vehicle, $V_{av}$ represents the average velocity of the vehicle, and $V_{\left(ina\right)}$ represents the inactivity of the vehicle (slowness or pause).

(42) $$V_{av}=\frac{\sum_{i=0}^{n}V_{i}}{V_{t}}\;$$

During vehicle operation, instances of inactivity may occur due to traffic or road work. The vehicle’s inactivity can be determined as follows:

(43) $$V_{\left(ina\right)}=\frac{\sum_{i=0}^{n}V_{eina\left(t\right)}}{V_{t}}$$

where $V_{eina\left(t\right)}$ represents the expected inactive or paused time of the vehicle.

**Hypothesis** **2.** *Applying blockchain technology to the final layer of the CNN model enhances its security and improves malicious vehicle detection.*

**Proof.**  We have a set of all vehicles described as follows:

(44) $$\;\;V=\left\{V_{1},\;V_{2},\;.\;.\;.,\;V_{n}\right\}$$

The blockchain layer is added to the system to identify any suspected malicious vehicle as follows:

(45) $$V_{i}\in \;Vs\;\;$$

After acquiring the multi-labels as input, they are transmitted to the CNN, along with the encoder and decoder layers. Once the algorithms have decoded all the multi-labels, the original multi-labels are sent to the SPP layer. □

## 6. Experimental Results

To validate the performance of the proposed CBCNN, realistic testing scenarios for detecting malicious vehicles are generated, and the proposed approach is compared to state-of-the-art approaches, namely SCNN [31], PPCDA [37], and multi-label classification with label graph superimposing (MCLGS) [38]. These tests are conducted to determine accuracy, malicious label detection, and loss ratio for realizing practical outcomes. We simulated the traffic from Taepyeong-dong in Gyeonggi Province, South Korea, to Samseong-dong in Seoul, South Korea, 14.4 KM, in order to establish a realistic testbed. The proposed approach is validated using C++ and implemented on ns-3.34. Vehicle modeling is performed using a concept illustrated in [39]. Multiple scenarios comprising both malicious and safe multi-labels are used to perform the testing process. The multi-labels are transferred to the vehicles using the proposed CBCNN, which analyzes the multi-labels. Notably, the network generated by vehicles does not require any infrastructure, and vehicle-to-vehicle communication can be established without the need for a physical conduit or a centralized regulating authority. Switches and hubs are not necessary in a VANET, as hop-to-hop communication is available.

IoT devices face several challenges and limitations in terms of mobility and scalability. The energy-efficient border node medium access control (BN-MAC) protocol is used to address these problems [40]. The primary objective of implementing BN-MAC is to reduce energy consumption while maintaining high levels of mobility, scalability, and collision avoidance at the second layer. Several testing scenarios were developed, starting with 60 vehicles. Despite the large number of vehicles, there was minimal traffic congestion. Consequently, the number of multi-labels for vehicles was increased in each testing scenario; therefore, the proposed vehicular network could accommodate up to 18,000 multi-labels for vehicles, exhibiting a realistic situation. The performance of CBCNN and its counterparts is evaluated, and the minimum system requirements are listed in Table 2.

The following notable outcomes were revealed through the experiments:

▪ Malicious vehicle detection.
▪ Average accuracy.
▪ Multi-label Loss.
▪ Cost/KWh reduction in energy consumption.

### 6.1. Datasets

NS-3 makes use of the data collection framework (DCF) to collect the datasets, which allows it to obtain data generated by simulator models, execute online reduction and data processing, and translate data into multiple output formats.

The DCF is divided into three primary classes: probes, collectors, and aggregators. The probe is a mechanism for instrumenting and controlling the simulation data output, which is used to monitor occurrences. It generates one or more ns-3 trace sources as output. Probes are connected to one or more trace sinks (known as Collectors), that analyze samples online and arrange them for output. The collector, on the other hand, consumes the data generated by one or more probe objects. It transforms data by performing operations such as normalization, elimination, and calculations of fundamental statistics. Collector objects do not directly send data to the ns-3 run; rather, they output data downstream to another sort of object known as an aggregator.

The data acquired by a network of probes and collectors is routed to the aggregator. The aggregator’s primary function is to marshal data and associated metadata into various output forms, such as plain text files, databases, and spreadsheet files. The raw data for vehicle detection is obtained from the following two main sources:

▪ The KITTI benchmark dataset [41].
▪ Tsinghua-Tencent Traffic-Sign Dataset [42].

**Figure 4:**
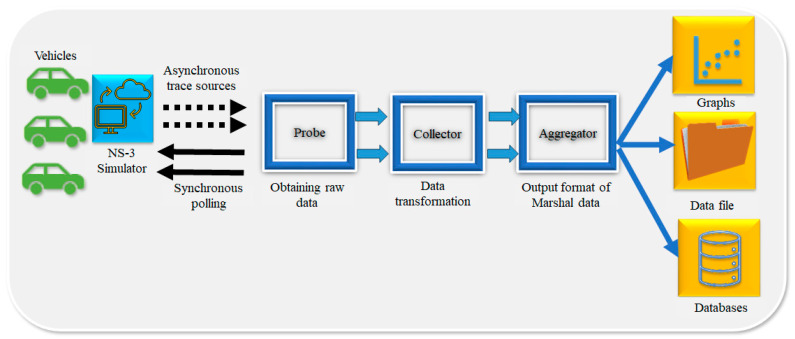
Dataset storage process through trace sources and polling.

The dataset storage process is depicted in Figure 4.

### 6.2. Malicious Vehical Detection

**Figure 5:**
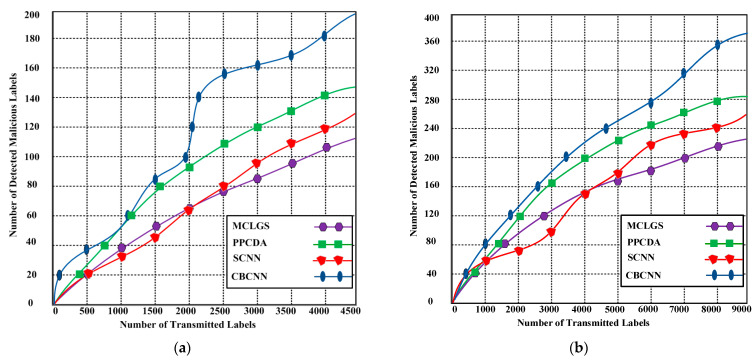
(**a**) Detected malicious labels with the proposed CBCNN and competing methods using a maximum of 4500 transmitted labels; (**b**) Detected malicious labels with the proposed CBCNN and competing methods using a maximum of 9000 transmitted labels.

The proposed CBCNN takes two or more labels as inputs to account for potential corruption by hackers. Its primary focus is on detecting suspected vehicle labels. The incorporation of consortium blockchain technology into our model yields clear results, as depicted in Figure 5. As illustrated in Figure 5a, the proposed CBCNN detects 200 malicious labels, whereas MCLGS, SCNN, and PPCDA detect 115, 128, and 146 malicious labels, respectively, out of 4500 transmitted labels. Figure 5b illustrates that the proposed CBCNN detected nearly 370 malicious labels, whereas MCLGS, CNNV, and PPCDA detected 222, 258, and 284 malicious labels, respectively, out of 9000 transmitted labels.

These results indicate that the proposed CBCNN demonstrated a higher level of success in utilizing consortium blockchain technology compared to the competing methods. To evaluate the performance of the multi-label system applied to vehicles, we must define the sets of false positives $F_{p}$, true positives $T_{p}$, and false negatives $F_{n}$, as the metrics used to assess these operations are dependent on the quantities of $F_{p}$, $T_{p}$, and $F_{n}$. Let β represent the set of bounding box predictions, γ represent the set of ground-truth bounding boxes, and $\left(P_{r},\varphi \right)$ represent the relationship between a prediction and ground-truth box on the same frame with $P_{r}\in \beta$ and φ∈γ. Consequently, we define the true positive $T_{p}$, false negative $F_{n}$, and false positive $F_{p}$ as follows:

(46) $$T_{p}=\left\{\left(P_{r},\varphi \right)|P_{r}\in \beta \;⋀\;\varphi \in \gamma ,⋀Ű\left(P_{r},\varphi \right)\geq D_{thr}\right\}$$

(47) $$F_{n}=\left\{\varphi |\varphi \in \gamma ,∄P_{r}\in \beta :\left(P_{r}\in \varphi \right)\in T_{p}\right\}$$

(48) $$F_{p}=\left\{P_{r}|P_{r}\in \beta ,∄\varphi \in \gamma :\left(P_{r}\in \varphi \right)\in T_{p}\right\}\;$$

where $Ű\left(P_{r},\varphi \right)$ denotes the connection over integration accumulated between the ground-truth φ∈γ and boundary boxes of the prediction $P_{r}\in \beta$, and $D_{thr}$ represents the detection threshold.

### 6.3. Average Accuracy

**Figure 6:**
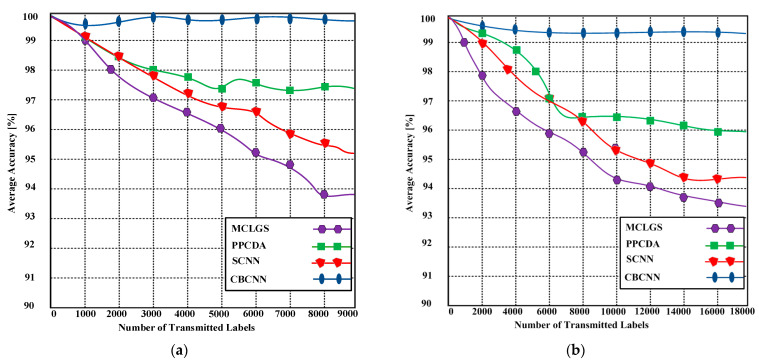
(**a**) Average accuracy of the proposed CBCNN and competing methods with up to 9000 transmitted labels; (**b**) Average accuracy of the proposed CBCNN and competing methods with up to 18,000 transmitted labels.

The average accuracy of detecting malicious labels serves as an assessment metric for evaluating the performance of CBCNN. The average accuracy varies depending on the number of transmitted labels, as illustrated in Figure 6. The experimental results demonstrate the superior accuracy of CBCNN. As illustrated in Figure 6a, CBCNN achieves an average accuracy of 99.84%, whereas MCLGS, PPCDA, and SCNN achieve average accuracies of 93.78%, 96.36%, and 95.19%, respectively, when utilizing 9000 transmitted labels. Furthermore, Figure 6b demonstrates that the proposed CBCNN achieves an average accuracy of 99.39%, whereas MCLGS, PPCDA, and SCNN achieve average accuracies of 93.38%, 95.96%, and 94.41%, respectively, when 18,000 transmitted labels are used. The utilization of blockchain technology is the primary factor contributing to the increased accuracy of the CBCNN.

### 6.4. Multi-Label Loss

**Figure 7:**
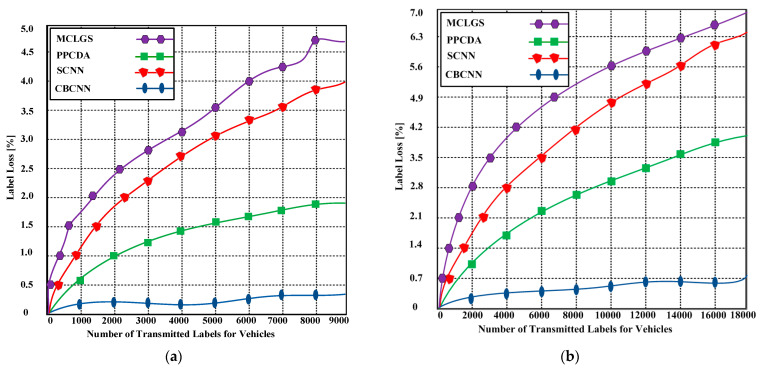
(**a**) Multi-label loss ratio of the proposed CBCNN and competing methods with 9000 transmitted labels; (**b**) Multi-label loss ratio of the proposed CBCNN and competing methods with 18,000 transmitted labels.

The multi-label loss predicts the effectiveness of the proposed method. Figure 7 illustrates the performance of the proposed CBCNN compared to other state-of-the-art approaches. The proposed technique exhibits a minimal label loss ratio of 0.32% with 9000 transmitted multi-labels, as depicted in Figure 7a.

In contrast, the competing techniques demonstrate label loss ratios of 1.89–4.69%. As the number of multi-labels increases to the level depicted in Figure 7b, the proposed approach maintains a loss ratio of 0.68% with 18,000 labels, whereas the competing strategies exhibit comparatively higher loss ratios of 4.04–6.52%. The key reason for the marginal loss in our approach is the utilization of four layers to accurately convey the labels while preventing label modification.

### 6.5. Cost/KWh Reduction in Energy Consumption

**Figure 8:**
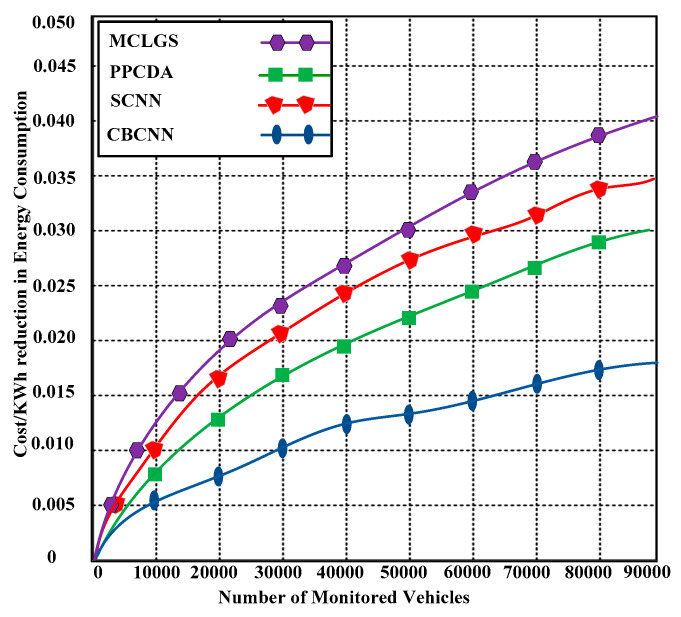
Cost/KWh reduction in energy consumption with 90,000 monitored vehicles.

The cost reduction has a positive impact on the economy and shows the trade-off between reduction and efficiency. In this experiment, the practical implications of the problem formulation from Equations (1)–(9) have been validated. Figure 8 demonstrates that the proposed CBCNN substantially reduced the cost, which is calculated at 0.0124 cost/KWh, whereas the contending methods produce 0.030 to 0.040 cost/KWh, which is much higher than the proposed CBCNN. Let $O_{C}$ be the original consumption of the power in KWh for the total number of the vehicles $V_{t}$, and after the end of counting the $V_{t}$, the new power consumption for $V_{t}$, is $N_{c}$. Thus, the total cost reduction $C_{rt}$ can be calculated as follows:

(49) $$C_{rt}=\left\{\left(\frac{\left(O_{C}-N_{c}\right)}{O_{C}}\right)\;V_{t}\times 100\right\}\;$$

## 7. Discussion of Result

The analysis of the experimental outcomes yielded intriguing observations. Evidently, the most effective approach for multi-object tracking in autonomous driving scenarios is the deployment of CBCNN with a multi-label detector. However, even for the best-performing methods, there is still significant room for improvement in absolute performance. Therefore, further enhancements may be required for the multi-object detectors used in autonomous driving situations. It should be noted that if the missed detections and incorrect classifications occur on distant objects from the vehicle, the aforementioned issues may be less critical. In fact, depending on the speed of the vehicle, braking or turning may not be necessary to avoid distant objects. The lack of consideration for the distance from objects in existing tracking measures and the absence of weighting mistakes based on that distance are drawbacks. Analyzing this factor could be crucial in selecting the most suitable strategy among different approaches. The proposed CBCNN employs various processes to detect malicious vehicles. Initially, the vehicle images are fed into the proposed CBCNN, which leverages the advantages of blockchain technology by connecting it to the final layer. In this layer, the multi-labels are stored as blocks, with each block containing the hash keys and public and private keys of their neighbors. Additionally, there is a feature detection component that includes all the features of the malicious blocks for matching, thereby enabling the identification of suspected, legitimate, and neutral vehicles. The assessment demonstrates that the proposed technique outperforms the state-of-the-art methods of MCLGS, SCNN, and PPCDA in terms of average accuracy and malicious label detection. The performance of the proposed CBCNN and the competing methods is presented in Table 3.

Future research should focus on selecting metrics that account for object distance and transforming loss functions utilized to train the multi-object detectors and the neural networks used for extracting object descriptors. Having labeled data with distance information would greatly assist this line of inquiry. Notably, none of the multi-object tracking techniques have been optimized to perform data association on objects belonging to multiple classes; rather, all the methods under consideration are aimed at carrying out the re-identification of a single class of objects, typically vehicles, and are only slightly modified for the multi-label. Therefore, developing data association algorithms that consider various object types is another area that warrants exploration. Furthermore, novel approaches to forecasting the future location of tracked objects should be investigated.

Another notable limitation is the lack of simulations of similar identification problems in driving scenarios to assess their impact on autonomous navigation. A framework that enables the integration of artificial vision algorithms and the simulation-based verification of the detrimental consequences of perception errors on autonomous navigation would be valuable for this purpose. However, developing such a framework would be challenging due to the navigation algorithm operating in the world reference system. To overcome this challenge, multi-sensor fusion algorithms must be defined to track the calculation of the speed and direction of vehicles on the road. Providing researchers with access to such a tool would significantly advance their studies in the field.

## 8. Conclusions and Future Works

This section summarizes the insight gained from the study and suggests areas for future research.

### 8.1. Conclusions

A four-layered paradigm called CBCNN is proposed for detecting malicious/non-malicious vehicles. The proposed CBCNN paradigm incorporates a CNN-enabled IoT model, geographic pyramid pooling, fixed-layer, and consortium blockchain technology. The primary objective is to detect malicious vehicles accurately while enhancing accuracy and reducing the loss ratio and cost. The proposed CBCNN approach demonstrates the effectiveness of using consortium blockchains for handling sequential data. The consortium blockchain incorporates a proof-of-luck mechanism, allowing vehicles to save energy while providing correct vehicle information to the vehicle management system. The CBCNN is tested using multi-label classification of up to 9000–18,000 transmitted labels without incurring additional costs. The results confirm that the proposed CBCNN provides higher malicious detection capability as compared to competing methods. The CBCNN detects 370 malicious labels by transmitting a maximum of 18,000 labels, which is a larger number than competing approaches. On the other hand, the competing approaches determine 222 to 286 malicious labels, which is 22.7 to 40% less. The proposed CBCNN obtains an accuracy of 99.84% with a maximum of 18,000 labels, which is 3.43–6.01% higher than competing approaches. The proposed CBCNN produces a lower loss ratio of 0.68%, while the loss ratio for competing approaches ranges from 4.04 to 6.52%. Furthermore, the proposed CBCNN has also been evaluated and tested for parameter tampering attacks, which exhibit outstanding resilience. The findings demonstrate that the proposed CBCNN is a superior paradigm for malicious vehicle detection, outperforming competing methods in terms of accuracy, malicious label detection, loss ratio, and cost reduction.

### 8.2. Future Works

In future research, data enrichment methods will be utilized to fine-tune various CNN models. Different quality-of-service metrics, including delay time, computational complexity, space complexity, and throughput, will be measured.

## Figures and Tables

**Table 1:** Contemporary work of existing approaches.

Works	Blockchain-Enabled Methods	Features	Vulnerabilities
Nasir et al. [31]	A blockchain-enabled convolutional neural network model for potential attacks	The blockchain is introduced to protect a convolutional neural network model from potential attacks	Restricted only to the CNN model and lacks accuracy
Hongyan et al. [32]	A multi-layer location sharing system based on blockchain for single point of failure	The suggested technique is also applicable in dynamic situations. An accumulator is used to enhance the scheme’s effectiveness in data refreshing and verification. Furthermore, the efficacy of the scheme is assessed using a formal security study based on Real-Or-Random	Failed to obtain efficiency
Shan et al. [33]	The blockchain model is analyzed in depth for vehicle detection	Employed blockchain technology to ensure security and preserve data	Limited only to smart contracts and their implementation
Zhang et al. [34]	Blockchain is employed in artificial intelligence to secure data sharing and automatically train the models	A blockchain is used to secure data exchange and autonomously train models. To enhance the security and reliability of facial datasets, blockchain technology is used in conjunction with VGGFace deep neural networks.	Focused only on data tampering
Kumar et al. [35]	Integration of blockchain technology and AI-deep neural network model for secure data sharing	The blockchain network stores and exchanges the trained model to disseminate information about new cancer patients. Updates about new cases of new patients are addressed using a decentralized blockchain, which tracks all types of patients’ histories.	An invalid data sample can jeopardize the classification accuracy of a model as it increases the time required for model training. It consumes more execution time as the deep learning models always require a large amount of resources such as computing power to train the model
Wang et al. [36]	Privacy-preserving federated learning system for ciphertext-level	Proposed privacy-preserving federated learning system which utilizes blockchain as the fundamental technology to secure data. It also provides model aggregation and model filtering. The Multi-Krum technology is enhanced and coupled with homomorphic encryption	Unable to achieve verifiability
Chen et al. [37]	A privacy-preserving cross-domain authentication system for VANETs	A secure blockchain-based privacy-preserving cross-domain verification solution for VANETs is proposed. The proposed method blends group signature with blockchain technology. The group signature approach is used to allow cross-domain vehicle verification	The group signature technique does not provide enough privacy. So, it limits the privacy protection
Our Solution	Layered-based paradigm for malicious vehicle detection	The CBCNN is employed to detect and restrict malicious vehicles to accomplish accurate malicious label identification while enhancing accuracy and decreasing loss ratio.	Limited to specific scenarios of vehicle detection

**Table 2:** Summarized testing parameters and system specifications.

Name of Parameters	Description
Simulator	ns-3.34
Number of maximum multi-labels	18,000
Transmission Range	45 m
Initial energy of the IoT device attached with vehicle	4 J
Size of vehicular network	1200 × 1200 m^2^
pause time	10 s
Simulation time	50 min
Data frame	256 bytes
Data Packet size	512 bytes
MAC protocol	BN-MAC
Tested algorithm	CBCNN
Mobility Model	Manhattan mobility model
Contending methods	SCNN, PPCDA and MCLGS
Mobility	0.1–15 m/s
HARD Disk free space	500 MB
RAM	2048 MB
Operating system	Windows 10
Processor	Intel(R) Core 2 Duo

**Table 3:** Comparison of findings obtained for CBCNN and competing techniques.

Methods	Malicious Label Detection (Max: 4500 Labels)	Malicious Label Detection (Max: 9000 Labels)	Average Accuracy (Max: 9000 Labels)	Average Accuracy (Max: 18,000 Labels)	Loss Ratio (Max: 9000 Labels)	Loss Ratio (Max: 18,000 Labels)
MCLGS	115	222	93.78%	93.38%	4.69%	6.52%
SCNN	128	258	95.19%	94.41%	3.49%	6.32%
PPCDA	146	284	96.36%	95.96%	1.89%	4.04%
Proposed CBCNN	200	370	99.84%	99.39%	0.32%	0.68%
